# Myeloproliferative Neoplasm Driven by *ETV6-ABL1* in an Adolescent with Recent History of Burkitt Leukemia

**DOI:** 10.3390/curroncol30070444

**Published:** 2023-06-21

**Authors:** Samuele Renzi, Fatimah Algawahmed, Scott Davidson, Karin P. S. Langenberg, Fabio Fuligni, Salah Ali, Nathaniel Anderson, Ledia Brunga, Jack Bartram, Mohamed Abdelhaleem, Ahmed Naqvi, Kassa Beimnet, Andre Schuh, Anne Tierens, David Malkin, Adam Shlien, Mary Shago, Anita Villani

**Affiliations:** 1 Division of Haematology/Oncology, The Hospital for Sick Children, Toronto, ON M4B 1B3, Canada; 2 Department of Pediatrics, Division of Pediatric Hematology/Oncology, CHUL-Laval, Laval University, Quebec City, QC G1V 4G2, Canada; 3 Laboratory Medicine Program, University Health Network, Toronto, ON M5G 2M9, Canada; 4 Program in Genetics and Genome Biology, The Hospital for Sick Children, Toronto, ON M5G 0A4, Canada; 5 Department of Paediatric Laboratory Medicine, The Hospital for Sick Children, Toronto, ON M5G 0A4, Canada; 6 Princess Máxima Center for Pediatric Oncology, 3584 CS Utrecht, The Netherlands; 7 Department of Pediatric Haematology and Bone Marrow Transplant, Leeds Teaching Hospitals, Leeds LS9 7TF, UK; 8 Department of Hematology, Great Ormond Street Hospital, London WC1N 3JH, UK; 9 Department of Paediatrics, University of Toronto, Toronto, ON M5S 1A1, Canada; 10 Department of Haematology, Princess Margaret Hospital, Toronto, ON M5G 2C1, Canada; 11 Department of Laboratory Medicine and Pathobiology, University of Toronto, Toronto, ON M5S 1A8, Canada

**Keywords:** pediatric malignancies, oncology, myeloproliferative syndrome

## Abstract

*ETV6-ABL1* gene fusion is a rare genetic rearrangement in a variety of malignancies, including myeloproliferative neoplasms (MPN), acute lymphoblastic leukemia (ALL), and acute myeloid leukemia (AML). Here, we report the case of a 16-year-old male diagnosed with a MPN, 7 months post-completion of treatment for Burkitt leukaemia. RNA sequencing analysis confirmed the presence of an *ETV6-ABL1* fusion transcript, with an intact, in-frame *ABL* tyrosine–kinase domain. Of note, secondary *ETV6-ABL1*-rearranged neoplastic diseases have not been reported to date. The patient was started on a tyrosine kinase inhibitor (TKI; imatinib) and, subsequently, underwent a 10/10 matched unrelated haematopoietic stem cell transplant. He is disease-free five years post-transplant. Definitive evidence of the prognostic influence of the *ETV6-ABL1* fusion in haematological neoplasms is lacking; however, overall data suggest that it is a poor prognostic factor, particularly in patients with ALL and AML. The presence of this ETV6-ABL1 fusion should be more routinely investigated, especially in patients with a CML-like picture. More routine use of whole-genome and RNA sequencing analyses in clinical diagnostic care, in conjunction with conventional cytogenetics, will facilitate these investigations.

## 1. Introduction

*ETV6-ABL1* gene fusion is a rare genetic rearrangement in a variety of malignancies, including myeloproliferative neoplasms (MPN), acute lymphoblastic leukemia (ALL), and acute myeloid leukemia (AML) [1]. This fusion results in enhanced tyrosine kinase activity of ABL1 and thus neoplastic transformation, producing molecular properties and mirroring the *BCR-ABL1* rearrangement [2,3].

Here, we report the case of a 16-year-old male diagnosed with a MPN, 7 months post-completion of treatment for Burkitt leukaemia. Detailed genomic analysis, including whole genome and transcriptome sequencing, was carried out through the SickKids Cancer Sequencing (KiCS) Program to further clarify the diagnosis of the MPN and its relationship to the original Burkitt leukemia.

## 2. Case Report

**Figure 1:**
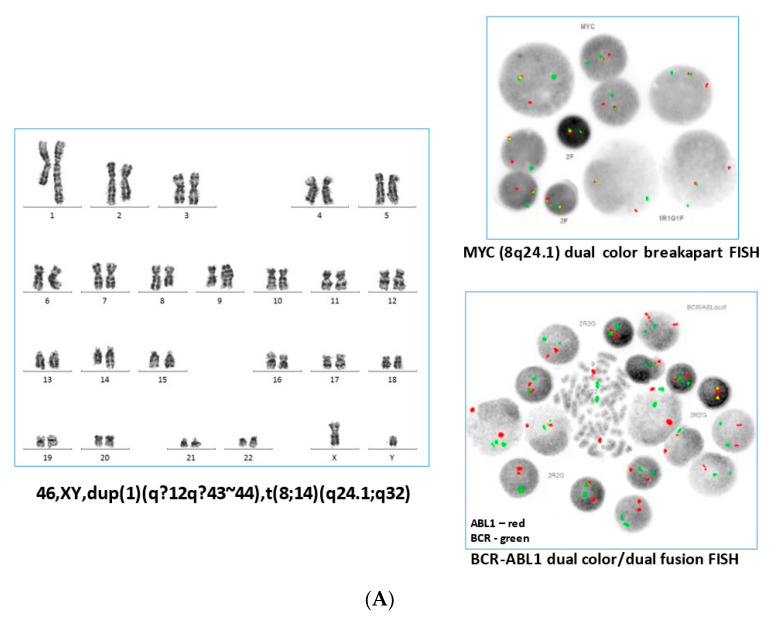

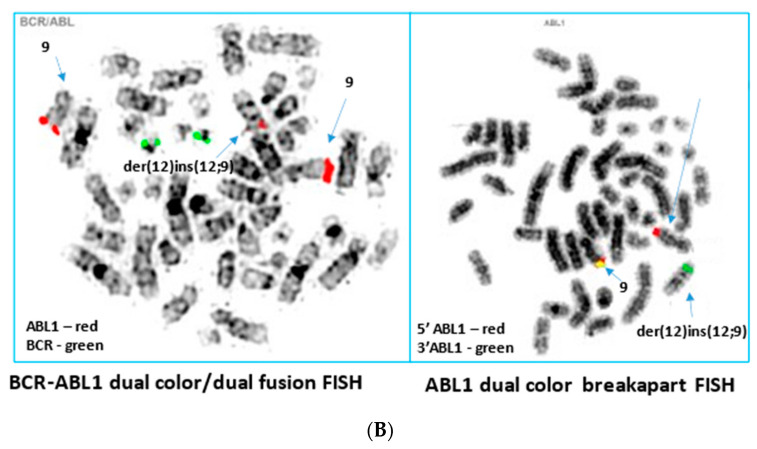
(**A**) Representative karyotype from the blood sample obtained at initial diagnosis with Burkitt Leukaemia (left panel), demonstrating a t(8;14) and duplication of 1q. Interphase FISH analysis with a dual colour breakapart probe for the *MYC* gene (8q24.1) showed *MYC* gene rearrangement in the majority of cells (upper right panel). After the patient subsequently presented with MPN, *BCR-ABL1* FISH performed on an archived Burkitt Leukaemia sample yielded normal results (lower right panel). (**B**) By G-band analysis of the myeloproliferative neoplasm, the karyotype appeared normal. Sequential *BCR*-*ABL1* metaphase FISH analysis on G-banded cells showed insertion of the *ABL1* signal into 12p13, with no involvement of the *BCR* gene (left panel). Metaphase FISH analysis using an *ABL1* breakapart FISH probe (right panel) showed the insertion of 3′*ABL1* into 12p13. Results of the *ETV6* breakapart as well as the subtelomeric 9q and 12p FISH testing were normal, consistent with an insertion mechanism rather than a translocation mechanism in the generation of the *ETV6-ABL1* fusion identified by molecular analysis.

The patient presented with a diffuse petechial rash, bilateral subconjunctival haemorrhages, and haemoptysis, on a background of a history of several months of fatigue, bone pain, and night sweats. In their family history, a maternal grandmother had died of pancreatic cancer at age 67, and a maternal uncle had been diagnosed with childhood leukaemia. Complete blood count (CBC) showed a white blood cell (WBC) count of 101 × 10^9^/L, haemoglobin at 106 g/L, and a platelet count of 12 × 10^9^/L. A peripheral smear showed 75% leukemic blasts. Immunophenotyping results can be found in Supplementary Data S1. Fluorescence in situ hybridization (FISH) analysis with a MYC breakapart FISH probe (Abbott Molecular, Abbott Park, IL, USA) was consistent with the presence of an *MYC* gene rearrangement in 170/200 (85%) cells, and G-band analysis showed an abnormal male karyotype of 46,XY,dup(1)(q12q43~44),t(8;14)(q24.1;q32)[18]/46,XY[2] (Figure 1A, left and upper right panel). A diagnosis of Burkitt leukaemia was made, and treatment was started according to the institutional standard of care (Supplementary Data S2).

A CBC 7 months after the completion of therapy showed WBC 29.2 × 10^9^/L, with a significant left shift, eosinophilia (2.92 × 10^9^/L), monocytosis (4.38 × 10^9^/L), thrombocytopenia (100 × 10^9^/L), and no peripheral blasts. The patient was asymptomatic. Viral workup was negative, and EBV serology was consistent with past infection. There was no travel history. Bone marrow aspirate (BMA) revealed marked hypercellularity with significant eosinophilia and an absence of elevated blasts consistent with a myeloproliferative disorder. Immunophenotyping showed no abnormal cell population. FISH analyses were negative for *MYC*, *PDGFRA* (Cytocell OGT, Cambridge), and *PDGFRB* (Cytocell OGT, Cambridge) gene rearrangements, and the karyotype was normal. Molecular analysis was negative for the *BCR-ABL1*, *FLT3*-ITD, *JAK2* V617F, and *CALR* variants.

The leucocytosis increased to 192 × 10^9^/L over 3 months, accompanied by persistent thrombocytopenia (range 50–100 × 10^9^/L). The patient became progressively symptomatic with fatigue, generalized bone pain, as well as significant spontaneous bruising. He was started empirically on Hydroxyurea 25 mg/kg/dose, which normalized his WBC count and improved his symptoms within one month.

**Figure 2:**
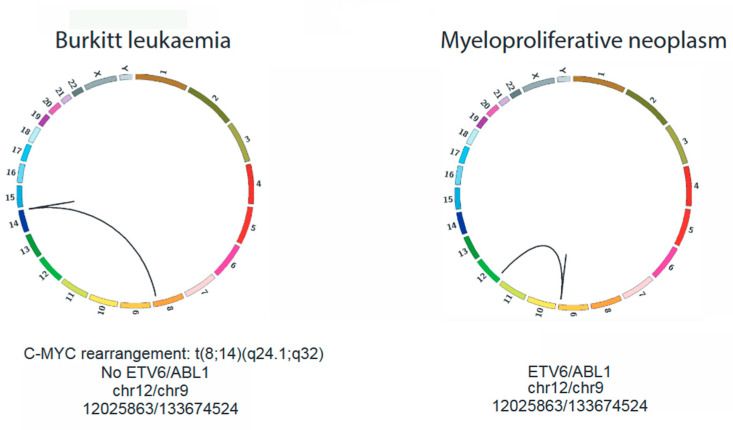
Circos plots generated from WGS of the Burkitt Leukaemia (**left**) and MPN (**right**) samples. Both samples represent diagnostic specimens (peripheral blood for the Burkitt leukaemia and bone marrow aspirate for the MPN). *ETV6-ABL1* rearrangement (chr12:12025863-chr9:133674524) was confirmed only in the MPN sample.

Whole-genome sequencing of his MPN BMA sample was performed on an Illumina HiSeqX platform with a depth of 22X, and RNAseq was performed on Illumina HiSeq 2500, with a depth of 294X. Structural variant analysis from whole-genome sequencing showed a dispersed duplication event of approximately 600 kb from ABL1 to PRRC2B (9q34), inserted into the ETV6 locus (12p13) (Figure 2, right panel). RNAseq analysis confirmed the presence of a corresponding *ETV6-ABL1* fusion transcript, with an intact, in-frame *ABL* tyrosine–kinase domain.

Cytogenetic re-evaluation of the MPN BMA sample using sequential FISH analysis with a *BCR-ABL1* probe (Cytocell OGT, Cambridge, UK) was suggestive of a cryptic insertion of a portion of the *ABL1* locus into one chromosome 12 at 12p13 (Figure 1B, left panel). *ABL1* gene rearrangement was present in 193/200 (96.5%) interphase cells. Interphase FISH analysis with a dual colour breakapart probe for the *ETV6* locus (Abbott Molecular, Abbott Park, IL, USA) did not show *ETV6* gene rearrangement in this patient, and metaphase FISH with subtelomeric probes for 9q and 12p yielded normal results, suggestive of the insertion of *ABL1* into *ETV6* rather than rearrangement by translocation. FISH analysis with a dual colour breakapart probe for *ABL1* (Cytocell OGT, Cambridge, UK) demonstrated that the 3′ portion of *ABL1* was inserted into 12p13 on the derivative chromosome 12, with the 5′ portion of *ABL1* remaining on the derivative chromosome 9 (Figure 1B, right panel). The karyotype was updated to 46,XY[20]. ish ins(12;9)(p13;q34q34)(12ptel+,3′ABL1+;5′ABL1+,9qtel+)[5].

We were interested in further exploring the relationship between the patient’s two neoplastic processes. The WGS performed on the diagnostic Burkitt leukemia blood sample did not show evidence of the *ETV6-ABL1* fusion (Figure 2, left panel). The RNA was not available for analysis. The results of the *BCR-ABL1* FISH analysis of 200 stored fixed cells from the Burkitt leukemia sample were also negative for the *ABL1* gene rearrangement (Figure 1A, lower right panel). The germline analysis by the WGS of the skin’s biopsy-derived fibroblasts did not reveal any pathogenic variants.

The patient was started on a tyrosine kinase inhibitor (TKI; imatinib) and, subsequently, underwent a 10/10 matched unrelated haematopoietic stem cell transplant. He is disease-free five years post-transplant.

## 3. Discussion

*ETV6-ABL1*-rearranged hematologic malignancies are rare entities [1]. To date, 51 cases have been reported [1,4,5]. The fusion was first described in a patient with ALL [6] and, subsequently, in patients with AML [7] and “CML-like” disease [8]. *ETV6-ABL1* is found in <1% of all children and adults diagnosed with ALL and is less frequently in AML. The exact incidence of CML-like *ETV6-ABL1* neoplasms is unknown, due to the lack of systematic screening [1].

Conventional clinical diagnostic tests often fail to detect this fusion, given that there is no commercial *ETV6-ABL1* FISH probe available, and the commonly used *BCR-ABL1* or *ETV6-RUNX1* probes can miss *ETV6-ABL1* [9]. An *ABL1* breakapart FISH probe would better enable the detection of all the *ABL1* gene rearrangements, including *ETV6-ABL1*. RT-PCR analysis can also produce false negative results unless the exact breakpoints are known. RNAseq has been increasingly recognized as the most useful diagnostic tool for these cases [1].

The unique presentation of our patient and the fact that *ETV6-ABL1* aberration can present in the form of immature lymphoid or myeloid neoplasms raise a number of possible pathogenetic mechanisms. First, it is possible that this patient had independent but simultaneous bi-lineage disease with two clones: the *MYC*-positive Burkitt leukemia and the *ETV6-ABL1* MPN, the former being controlled by the initial Burkitt-targeted chemotherapy, allowing the other partially treated clone with *ETV6-ABL1* to expand following therapy completion. Of note, our patient had marked eosinophilia at the presentation of both the Burkitt leukemia (1.01 × 10^9^/L) and the *ETV6-ABL1* MPN (4.67 × 10^9^/L)—a finding that is commonly associated with MPNs and not with Burkitt disease [10]. Furthermore, FISH and WGS may have missed a subclone of *ETV6-ABL1* MPN in the Burkitt leukemia sample due to low sensitivity; the lack of RNA for in-depth RNAseq did not allow us to confirm or refute this hypothesis. Of note, no pathogenic variants were identified on germline analysis to suggest an underlying cancer predisposition syndrome, which must be considered in the setting of two independent primary tumors.

The second possible molecular mechanism is that the *ETV6-ABL1* was an early clonal event that occurred in a common hematopoietic progenitor cell, followed by a second hit in the *MYC,* resulting in the initial Burkitt leukemia. However, we would have expected the clonal presence of the *ETV6-ABL1* fusion in the diagnostic Burkitt leukemia specimen in this circumstance.

Alternatively, the MPN may have been a therapy-induced secondary event associated with the alkylator chemotherapy included in the treatment of Burkitt leukemia. However, secondary *ETV6-ABL1*-rearranged neoplastic diseases have not been reported to date. Furthermore, such an early secondary malignancy in a paediatric patient after exposure to alkylators would be very unlikely, as the latency is typically several years post-completion of therapy [11].

Definitive evidence of the prognostic influence of the *ETV6-ABL1* fusion in haematological neoplasms is lacking; however, overall data suggests that it is a poor prognostic factor, particularly in patients with ALL and AML [1,12]. There also are no definitive data to standardize the treatment for *ETV6-ABL1* MPNs, although multiple reports advocate for the early introduction of first and/or second generation TKIs [13,14,15]. Recently, Zhang et al. published a retrospective series of 42 adult patients with a confirmed myeloproliferative neoplasm associated with eosinophilia and a tyrosine kinase gene fusion. The study showed an excellent response to the upfront tyrosine kinase inhibition, regardless of the type of genetic rearrangement; however, none of the patients in this series had an *ETV6-ABL1* gene fusion [16]. It should also be noted that monotherapy with TKIs may result in transient responses: Brien et al. reported the case of a 38-year-old man with an *ETV6-ABL1*-driven chronic myeloid leukemia who initially responded to imatinib but who rapidly deteriorated over the following 2 weeks and required conventional chemotherapy [17]. The mechanisms responsible for TKI resistance remain unclear; Zimmermannova et al. have suggested that an activating mutation in the GNB1 gene could result in a resistance to TKI in the context of *ETV6-ABL1*-associated neoplasms, via the restoration of signalling through the phosphoinositide-3-kinase (PI3K)/Akt/mTOR and mitogen-activated protein kinase (MAPK) pathways [18]. With regard to other *ABL*-class fusions, such as *BCR-ABL1*, it is well-established that point mutations in the kinase domain of *BCR-ABL1* are the prevalent mechanisms of resistance to ABL kinase inhibition (60% of cases; termed “*BCR-ABL1* dependent resistance”) [19]. In other cases, other factors have been postulated, such as the activation of alternative signalling pathways, including the RAS/MAPK and JAK/STAT pathways, or changes in epigenetic regulation (termed *BCR-ABL1* independent resistance) [20,21].

Further detailed molecular studies of other patients with the same genomic abnormalities will provide insight into the pathogenesis and clinical course of *ETV6-ABL1*-associated neoplasms. The presence of this fusion should be more routinely investigated, especially in patients with a CML-like picture, particularly as it can prompt therapy with a tyrosine kinase inhibitor, as in the patient presented here. More routine use of WGS and RNAseq analysis in clinical diagnostic care, in conjunction with conventional cytogenetics, will facilitate these investigations.

## Data Availability

This being a case report, data are confidential.

## Supplementary Materials

The following supporting information can be downloaded at: https://www.mdpi.com/article/10.3390/curroncol30070444/s1, Data S1: Immunophenotype of Burkitt’s Leukaemia; Data S2: Chemotherapy received for treatment of Burkitt’s Leukemia, based on institutional standard of care for B-cell Non-Hodgkin Lymphoma, group C.
