# Supplementary material for: Myeloproliferative Neoplasm Driven by ETV6-ABL1 in an Adolescent with Recent History of Burkitt Leukemia
Source: Curr Oncol. 2023 Jun 21;30(7):5946–52. doi: 10.3390/curroncol30070444 (PMC10378670; doi:10.3390/curroncol30070444)
Supplement: Supplementary file 1 [file curroncol-30-00444-s001.zip › curroncol-2403660-supplementary.pdf]

**Supplemental Data**

**Supplement S1:** Immunophenotype of Burkitt's Leukaemia
Immunophenotyping of these cells revealed them to be an abnormal population of B-lymphocytes defined by lambda-restricted mature B-cells (positive for CD10, CD19, CD20, CD38, cCD22 cCD79a, CD45, cIgM, CD9, CD19+Lambda+, and negative for TdT, CD34, CD19+kappa+, CD2, CD3, CD4, CD5, CD7, CD8, CD33, CD13, CD15, CD123, CD66c, CD73, cCD3, cMPO, TSLPR).

**Supplement S2**: Chemotherapy received for treatment of Burkitt's Leukemia, based on institutional standard of care for B-cell Non-Hodgkin Lymphoma, group C
This patient received 1 course of COP reduction (cyclophosphamide, vincristine, prednisone), 2 cycles of R-COPADM (rituximab, cyclophosphamide, vincristine, prednisone, doxorubicin, high-dose methotrexate), followed by 2 courses of R-CYVE (Rituximab, high-dose cytarabine, etoposide) and 2 courses of maintenance (high-dose methotrexate, vincristine, prednisone, cyclophosphamide, doxorubicin, cytarabine, etoposide). He also received CNS-directed intrathecal chemotherapy.
